# Comparison of risk of stroke in patients treated with peritoneal dialysis and hemodialysis: a systematic review and meta-analysis

**DOI:** 10.1080/0886022X.2019.1632210

**Published:** 2019-07-11

**Authors:** Xiaojiang Zhan, Mei Yang, Yanbing Chen, Li Zhang, Caixia Yan, Yu Wang

**Affiliations:** Department of Nephrology, The First Affiliated Hospital of Nanchang University, Nanchang, Jiangxi, China

**Keywords:** Peritoneal dialysis, hemodialysis, stroke, meta-analysis

## Abstract

**Objective:** Accumulating evidence has demonstrated that dialysis patients are at increased risk for stroke. However, the impact of dialysis modalities on stroke risk remains controversial. We conducted a systematic review and meta-analysis to determine the effect of peritoneal dialysis (PD) and hemodialysis (HD) on stroke risk.

**Methods:** A systematic search of PubMed, EMBASE, and Web of Science was performed to identify articles comparing the stroke outcomes of dialysis patients. Hazard ratios (HRs) with 95% confidence intervals (95% CIs) were extracted and synthesized to examine stroke outcomes, including ischemic stroke, hemorrhagic stroke, and overall stroke.

**Results:** The search yielded five studies composed of 1,219,245 patients that were evaluated in the final analysis. The results showed that PD was associated with a lower risk for hemorrhagic stroke compared with HD (HR = 0.78; 95% CI: 0.69–0.88; *p* < 0.001). For ischemic stroke, the results showed that PD was associated with a higher risk compared with HD among the non-Asian patients (HR = 1.13; 95% CI: 1.05–1.23; *p* = 0.002), but there were no significant differences between PD and HD for the Asian patients. Similarly, there were no significant differences between the effects of the PD and HD approaches on overall stroke risk.

**Conclusions:** We observed that PD patients were less likely to develop hemorrhagic stroke than HD patients, and the risk for ischemic stroke was significantly higher for PD patients than for HD patients among the non-Asian patients. However, our findings could be biased due to the heterogeneity of the included studies.

## Introduction

The global prevalence of end-stage renal disease (ESRD) has increased sharply in recent years. Hemodialysis (HD) and peritoneal dialysis (PD) have been widely accepted for treatment of ESRD [1]. Cardiovascular disease (CVD) is the most common cause of morbidity and mortality in ESRD patients in whom dialysis therapy is initiated, accounting for 33% of hospitalizations, 37% of rehospitalizations, and 41% of deaths [2]. Stroke represents one of the main causes of cardiovascular mortality in patients with ESRD [3,4]. PD has been considered to be superior to HD for cerebrovascular protection because anticoagulation is not required during PD, and PD maintains better control of hypertension. However, PD patients may experience altered glucose metabolism, hypervolemia and exposure to glycated end products from the dialysate, which promote arteriosclerosis [5].

There are few studies comparing stroke risk in PD versus HD patients, and the results are conflicting. We, therefore, performed a meta-analysis of the available published literature to compare the effect of the two modalities on stroke risk.

## Methods

### Literature search

A literature search of PubMed, EMBASE, and Web of Science was performed to identify relevant studies. No date or language restrictions were applied. The search terms included ‘stroke’ ‘intracranial embolism’ ‘cerebral infarction’ ‘brain infarction’ ‘ischemic attack’ ‘cerebrovascular disease’ ‘cerebrovascular disorder’ ‘hemorrhagic stroke’ ‘cerebrovascular accident’ ‘peritoneal dialysis’ ‘hemodialysis’ ‘haemodialysis’ ‘renal insufficiency, chronic’ ‘renal dialysis’ and ‘kidneys, artificial’. The Boolean operators ‘OR’ and ‘AND’ were used to facilitate the search. The reference lists of retrieved articles were manually searched to identify related articles. The latest date of this search was 30 June 2018.

### Inclusion criteria

The following inclusion criteria were used: (1) the study design was a cohort study that evaluated the association between the dialysis modality (PD or HD) and stroke; (2) the outcomes of interest were stroke events; and (3) the hazard ratios (HRs) with 95% confidence intervals (CIs) were provided.

### Exclusion criteria

The following exclusion criteria were used: (1) the inclusion criteria were not met; (2) the study population included pediatric patients; (3) the method of data collection was not valid; and (4) the studies were duplicate articles, reviews, commentaries, or editorials.

### Data extraction

Two reviewers (X.Z. and M.Y.) independently extracted data from the included studies, and any discrepancy was resolved by discussion until a consensus was reached. The following information was extracted from each included study: the first author’s name, country of the population studied, year of publication, study design, inclusion and exclusion criteria, sample size, follow-up duration, characteristics of the study population, stroke events recorded, adjusted confounding variables, and study quality. In all cases of missing or incomplete data, the corresponding authors were contacted.

### Statistical analysis

This systematic review was performed according to the recommendations of the Cochrane Collaboration and the Quality of Reporting of Meta-analyses (QUORUM) guidelines [6,7]. Study heterogeneity was assessed using the chi-squared test with significance set at *P* < 0.10 and the *I*^2^ statistic. If *I*^2^ was >50%, a random-effects (RE) model was used. Otherwise, a fixed-effects (FE) model was used [8]. Subgroup analyses were used to explore the sources of heterogeneity. Sensitivity analyses were performed by removing individual studies one at a time to assess the robustness of the results. The Newcastle–Ottawa Scale (NOS), with some modifications to match the needs of this study, was used to assess the quality of the study and risk for bias [9,10]. This modified NOS consisted of three parts: the selection of the study patients, the comparability of the study groups, and the ascertainment of outcomes. A score of 0 to 9 stars was allocated to each study. Studies achieving a score of ≥ 6 stars were considered to be high quality. Meta-regression analysis was not performed due to the limited number of studies. Publication bias was evaluated using funnel plots. Statistical analysis was performed using Review Manager Version 5.3 (The Cochrane Collaboration, Oxford, London, UK). Generally, the results with a *P* values <0.05 (*α* = 0.05) were considered statistically significant.

## Results

### Description of eligible studies

**Figure 1.**
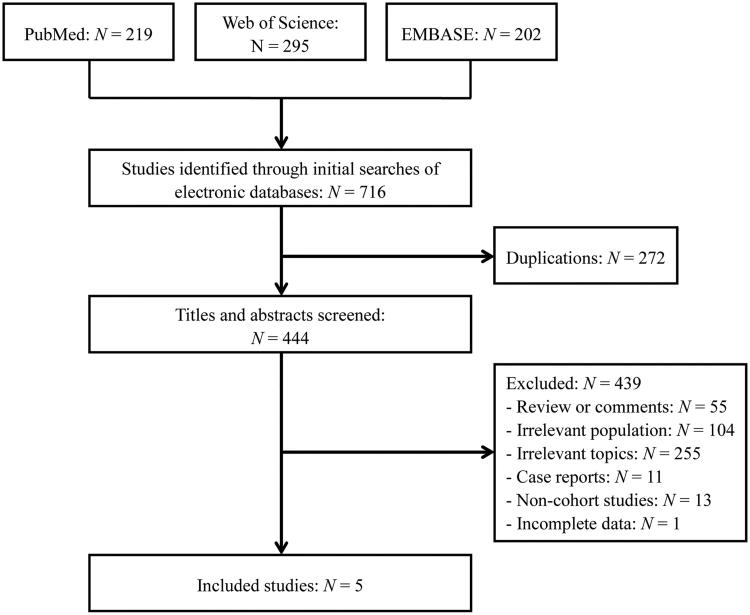
Flow diagram of studies identified, included and excluded.

Five studies [2,3,11–13] published from 2014 to 2016 fulfilled the inclusion criteria and were included in the meta-analysis (Figure 1). Examination of the reference lists of these studies did not identify any further studies for evaluation. Three studies [2,11,12] assessed all-cause stroke; five studies [2,3,11–13] assessed ischemic stroke; and four studies [2,3,11,12] assessed hemorrhagic stroke risk between the two groups. The characteristics of included studies are shown in Table 1. One study was a prospective cohort study; three studies were retrospective cohort studies; and the remaining one was a conference abstract. Two of these studies were conducted in China; one study was conducted in Korea; and the other two studies were conducted in Australia and the United States.

**Table 1.** Characteristics of included studies.

First author, year	Country	Design	No. of patients: PD/HD	Follow-up	Study quality (score)
Masson, 2016 [11]	Australia	Retrospective	3042/7422	3.8 (1.6–7.8)(m)^a^	★★★★★★★★
Kim, 2015 [2]	Korea	Retrospective	7387/22 892	21.5 (0–57)(m)^b^	★★★★★★
Fu, 2015 [12]	China	Prospective	305/285	32.5 (3–71.8)(m)^b^	★★★★★★★★
Stack, 2015 [13]	America	Retrospective	86 168/1,011,578	NA	★★★★
Wang, 2014 [3]	China	Retrospective	5974/74 192	HD: 4.2 ± 3.2（y）^c^	★★★★★★★
				PD: 3.0 ± 2.3（y）^c^	

a Interquarter range.

b Range.

c Mean ± standard deviation.

m: month; y: year; NA: data not available

### Methodological quality of included studies

Agreement between the two reviewers for study selection was 100%, and the quality assessment was 80%. For the included studies, the risk for bias was evaluated using the modified NOS.

### Association between dialysis modality and stroke risk

**Figure 2.**
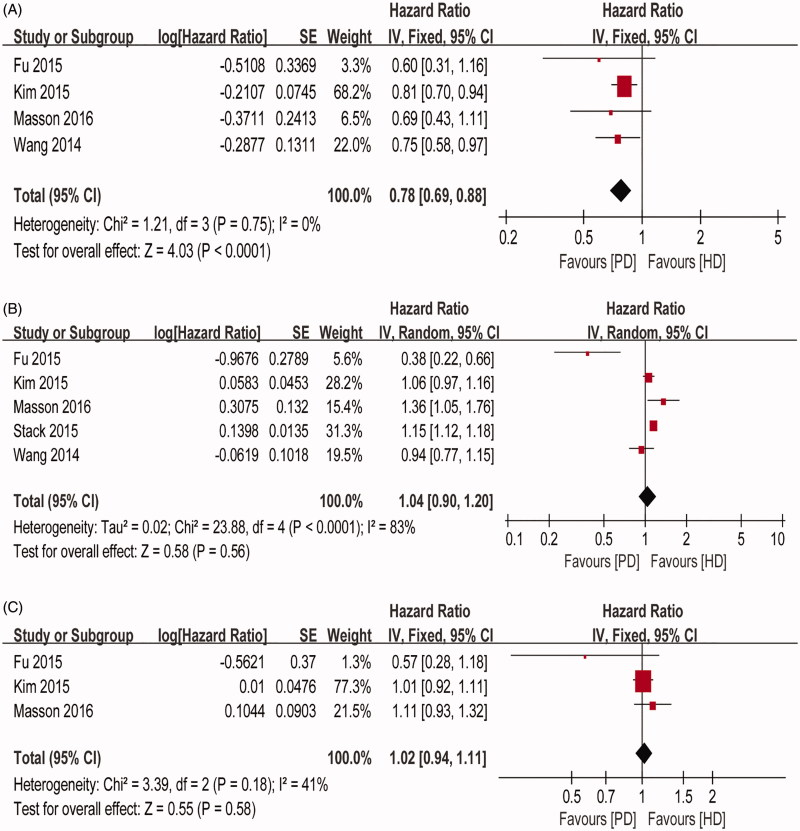
Meta-analysis of stroke risks comparing PD with HD: (A) hemorrhagic stroke; (B) ischemic stroke; and (C) overall stroke.

Three studies investigated the association between dialysis modality and overall stroke risk. The HR of stroke derived from individual studies and the pooled HR for PD versus HD patients are presented in Figure 2. The pooled data showed no significant difference between PD and HD patients in overall stroke risk (pooled HR = 1.02; 95% CI: 0.94–1.11; *P* = 0.58). There was no considerable heterogeneity among the studies (*P* = 0.18, *I*^2^ = 41%); therefore, we used a FE model for the analysis.

Five studies reported ischemic stroke risk. The individual studies and the pooled HR with a 95% CI for stroke in PD versus HD patients are shown in Figure 2. There were no significant differences between PD and HD patients in ischemic stroke risk (pooled HR = 1.04; 95% CI: 0.90–1.20; *P* = 0.56). Heterogeneity was present among these studies (*P* < 0.0001, *I^2^* = 83%). For the purpose of acquiring more conservative results, we chose a RE model for the analysis.

The pooled data from the four studies that reported hemorrhagic stroke risk showed that PD patients had a 22% lower risk of hemorrhagic stroke (pooled HR = 0.78; 95% CI: 0.69–0.88; *P* < 0.001) compared to HD patients, with no evidence of heterogeneity (*P* = 0.75, *I*^2^= 0%; Figure 2).

### Subgroup analyses

**Figure 3.**
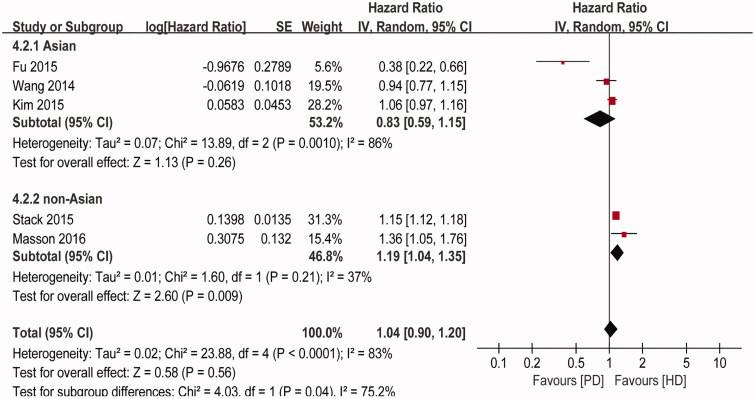
Subgroup analyses of ischemic stroke comparing PD with HD.

Because the ischemic stroke risk between PD and HD had high heterogeneity, we used subgroup analyses to examine the heterogeneity. A significant difference in the multivariable-adjusted HR of the ischemic stroke risk was observed between subgroups stratified according to region. Stratification based on region indicated that the pooled risk for ischemic stroke was significantly different in studies with non-Asian patients (pooled HR = 1.19, 95% CI: 1.04–1.35, *P* = 0.009). However, no association was identified in studies with Asian patients, and the difference was not statistically significant (*P* = 0.26) (Figure 3).

### Publication bias assessment and sensitivity analysis

**Figure 4.**
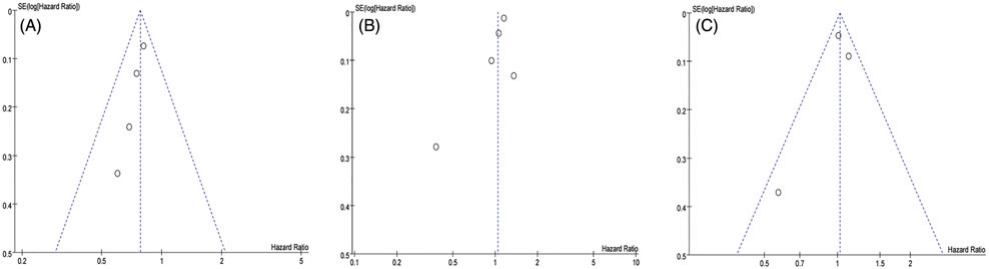
Funnel plots of (A) hemorrhagic stroke; (B) ischemic stroke; (C) overall stroke in our study.

We assessed publication bias with funnel plots. The funnel plots for overall stroke, ischemic stroke, and hemorrhagic stroke all showed slight asymmetry, suggesting possible publication bias (shown in the supplemental file for Figure 4). The sensitivity analysis of the ischemic stroke risk indicated that the omission of any of the studies changed estimates between 0.95 (95% CI: 0.72–1.24) and 1.11 (95% CI: 1.01–1.21) (Table 2). Deletion of the Fu et al. study decreased the heterogeneity from 86% to 64%, and there were significant differences between PD and HD patients and their ischemic stroke risk (pooled HR = 1.11; 95% CI: 1.01–1.21; *P* = 0.03), which was contrary to the original result.

**Table 2.** Sensitivity analysis of the ischemic stroke risk.

Study omitted	HR	95%CI		*I*^2^ (%)	*p*
Masson et al. (2016) [11]	0.99	0.85	1.16	86	.91
Kim et al. (2015) [2]	0.97	0.75	1.27	86	.84
Wang el al. (2014) [3]	1.07	0.91	1.25	85	.42
Stack et al. (2015) [13]	0.95	0.72	1.24	84	.68
Fu et al. (2015) [12]	1.11	1.01	1.21	64	.03

HR: hazard ratio; CI: confidence interval

## Discussion

This meta-analysis included 5 studies investigating the effects of dialysis modalities on stroke risk. After summarizing the available data from all the included studies, compared with HD patients, PD patients had a 22% lower risk of hemorrhagic stroke later in life.

ESRD patients have a 3–10 times greater risk of having a stroke compared with the general population [11]. The effect of traditional cardiovascular risk factors in ESRD patients may be different from that in the general population. For example, a previous study showed that among patients with ESRD and atrial fibrillation (AF), the incidence of stroke is significantly higher than in patients with ESRD who do not have AF [14]. Furthermore, treatments for ESRD (HD or PD) may have different effects on the risk for stroke. Kim et al. [2] described that PD patients had a 19% lower risk of hemorrhagic stroke than HD patients (HR = 0.81; 95% CI: 0.70–0.93). Wang et al. [3] showed that PD patients had a 25% lower risk of hemorrhagic stroke than HD patients (HR = 0.75; 95% CI: 0.58–0.96). Unfortunately, two other studies [11,12] did not find a significant difference between the two groups’ risk for hemorrhagic stroke. The current meta-analysis indicated that PD patients had a significantly lower risk for developing hemorrhagic stroke than HD patients, based on a pooled HR of 0.78 (95% CI: 0.69–0.88). This finding is consistent with the studies of Kim et al. [2] and Wang et al. [3]. There are several reasons to hypothesize why PD patients may have a lower risk for hemorrhagic stroke compared with HD patients. First, HD is marked by hemodynamic instability, leading to both hypotension and even hypertension during dialysis [15,16]. Repeated changes in blood pressure may predispose patients to hemorrhagic stroke. Second, HD patients also frequently receive anticoagulation as part of their treatment, which may increase the risk for hemorrhagic stroke [17].

There was high heterogeneity in the ischemic stroke risk for PD versus HD. Almost 10% of people in Australia are Asian, and almost 5% of people in the United States are Asian. Our subgroup analyses by region, which may have been a potential source of heterogeneity, revealed that heterogeneity was almost removed in the subgroup where there were only non-Asian patients. The exclusion of Fu et al.’s study noticeably decreased the heterogeneity, and the results changed. This study might have also played a partial role in the heterogeneity. Data on how the risks for ischemic stroke vary in PD and HD patients is inconsistent. Masson et al. [11] showed that PD patients are more likely to develop ischemic stroke than HD patients. There are three potential explanations. First, higher levels of pro-coagulant proteins and hemoconcentration are found in people undergoing PD compared to those undergoing HD [18]. Second, people who prefer PD may also have pro-thrombotic comorbidities, including heart failure, atherosclerotic heart disease, vascular access problems, or a previous stroke [19]. Third, most patients receiving PD use glucose-based dialysate, which may increase the burden of glucose and lead to more metabolic side effects, including obesity, dyslipidemia, hyperinsulinemia and peripheral insulin resistance, more than those receiving HD [20–22]. However, Fu et al. [12] reported that HD patients had a significantly higher risk for ischemic stroke compared to PD patients. A possible reason is that HD patients are more likely to exhibit characteristics that could increase the risk for stroke, such as an older age; the presence of comorbidities, such as diabetes, hypertension, and CVD; and worse nutritional status and residual renal function [12].

Studies comparing the risk for overall stroke in PD and HD patients have shown conflicting results [12,13], probably due to differences in the study populations (incident or prevalent dialysis patients) or methodology. Many previous studies have demonstrated that PD is noninferior to HD in the first two years. After that period, HD tends to be superior to PD [23,24]. The relative advantage of PD over HD in the early period is usually explained by better preservation of residual renal function or avoidance of complications associated with temporary vascular access in HD patients [25,26]. However, the early survival benefit of PD is difficult to maintain and is usually reversed over time. It is speculated that this reversal is due to an increase in the prevalence of dyslipidemia caused by exposure to large amounts of dialysate glucose in PD patients compared with those on HD [27] or by worsened cardiac remodeling due to excessive fluid overload accompanied by gradual deterioration of residual renal function and peritoneal ultrafiltration failure [28,29]. On the other hand, PD patients are more likely to shift to HD, so the methodology used may affect the results, as shown by Stack et al. [13]. To the best of our knowledge, the present study is the first meta-analysis that evaluated the association between dialysis modality and stroke risk. We applied a variety of strategies to include studies and evaluate the quality of the studies to minimize the effects of heterogeneity. We set no language and date restriction to minimize publication bias. Therefore, this analysis provides the latest information in this area.

The present meta-analysis has the following limitations that must be taken into account. First, a cause-and-effect relationship could not be established because the results of the study were based on cohort studies. Second, the number of studies included was relatively small. Third, heterogeneity among studies comparing the risk for ischemic stroke was marked.

In conclusion, this meta-analysis found that PD patients had a lower risk for hemorrhagic stroke compared to HD patients. However, the risks for ischemic stroke and overall stroke did not differ between the two groups. Although the methodology we adopted is rigorous, we must consider the inherent limitations of the included studies, so the conclusions drawn from our results should be interpreted with caution. An accumulation of high quality, observational studies is necessary to further confirm our findings.
